# Risk factors for progression of pulmonary fibrosis: a single-centered, retrospective study

**DOI:** 10.3389/fmed.2024.1335758

**Published:** 2024-02-07

**Authors:** Jia-Jia Fan, Jin-Min Gu, Si-Yao Xiao, Ming-Yue Jia, Gui-Ling Han

**Affiliations:** ^1^Department of Pulmonary Disease, Sunsimiao Hospital, Shanxi, China; ^2^Institute of Clinical Medicine, Beijing University of Chinese Medicine, Beijing, China; ^3^National Center for Respiratory Medicine, Beijing, China; ^4^State Key Laboratory of Respiratory Health and Multimorbidity, Beijing, China; ^5^National Clinical Research Center for Respiratory Diseases, Beijing, China; ^6^Institute of Respiratory Medicine, Chinese Academy of Medical Sciences, Beijing, China; ^7^Department of Traditional Chinese Medicine for Pulmonary Diseases, Center of Respiratory Medicine, China-Japan Friendship Hospital, Beijing, China

**Keywords:** interstitial lung diseases, progressive pulmonary fibrosis, prognosis, risk factors, ILD

## Abstract

**Objective:**

This study aimed to identify clinical characteristics associated with the prevalence of progressive pulmonary fibrosis (PPF) in interstitial lung disease (ILD) and to develop a prognostic nomogram model for clinical use.

**Methods:**

In this single-centered, retrospective study, we enrolled ILD patients with relatively comprehensive clinical data and assessed the incidence of PPF within a year using collected demographics, laboratory data, high-resolution computed tomography (HRCT), and pulmonary function test (PFT) results. We used a training cohort of ILD patients to identify early predictors of PPF and then validated them in an internal validation cohort and subsets of ILD patients using a multivariable logistic regression analysis. A prognostic nomogram was formulated based on these predictors, and the accuracy and efficiency were evaluated using the area under the receiver operating characteristic curve (AUC), calibration plot, and decision curve analysis (DCA).

**Results:**

Among the enrolled patients, 120 (39.09%) cases had connective tissue disease-associated interstitial lung disease (CTD-ILD), 115 (37.46%) had non-idiopathic pulmonary fibrosis idiopathic interstitial pneumonia (non-IPF IIP), and 35 (11.4%) had hypersensitivity pneumonitis (HP). Overall, 118 (38.4%) cases experienced pulmonary fibrosis progression. We found that baseline DLco% pred (OR 0.92; 95% CI, 8.93–0.95) was a protective factor for ILD progression, whereas combined pneumonia (OR 4.57; 95% CI, 1.24–18.43), modified Medical Research Council dyspnea score (mMRC) (OR 4.9; 95% CI, 2.8–9.5), and high-resolution computed tomography (HRCT) score (OR 1.22; 95% CI, 1.07–1.42) were independent risk factors for PPF. The AUC of the proposed nomogram in the development cohort was 0.96 (95% CI, 0.94, 0.98), and the calibration plot showed good agreement between the predicted and observed incidence of PPF (Hosmer–Lemeshow test: *P* = 0.86).

**Conclusion:**

ILD patients with combined pneumonia, low baseline DLco% pred, high mMRC marks, and high HRCT scores were at higher risk of progression. This nomogram demonstrated good discrimination and calibration, indicating its potential utility for clinical practice.

## Introduction

Interstitial lung diseases (ILDs) are a group of disorders that affect the lung parenchyma (1). Progression occurs in almost all patients with idiopathic pulmonary fibrosis (IPF), and a large proportion of those with non-IPF forms of ILDs may also develop a progressive phenotype, a condition characterized by self-perpetuating fibrosis, worsening respiratory symptoms, impaired lung function, and increased mortality despite conventional treatment (2–6). The estimated median survival time from symptom onset to death for patients with progressive pulmonary fibrosis (PPF) is 61–80 months, depending on the underlying etiology (4–6). The diagnosis of PPF requires evidence of lung function decline and a combination of physiological, radiological, and symptomatic deterioration over time (6).

Recent clinical trials have shown that antifibrotic agents, such as nintedanib and pirfenidone, can slow down the rate of lung function decline in ILD patients with progression phenotype (7–9). Nintedanib is a tyrosine kinase inhibitor that blocks multiple pathways involved in fibrogenesis and has been approved for the treatment of IPF and other fibrosing ILDs with a progressive phenotype (7). Pirfenidone is a drug with anti-inflammatory, antioxidant, and antiproliferative effects, shown to be effective in IPF and other fibrosing ILDs with a progressive phenotype (8, 9). The results of these trials suggest that there may be a common mechanism of fibrosis in ILD patients who progress to end-stage disease. However, the risk factors and prognostic indicators for PPF in ILD were not well established. Identifying these factors could help clinicians monitor and prevent the progression of PPF in ILD patients.

In clinical practice, monitoring disease progression includes various components. Clinical and laboratory data had proposed a relative decline in pulmonary function, progression fibrosis in HRCT, and elevated serum Krebs Von den Lungen-6 (KL-6), predicting progression in specific types of ILDs based on changes in variables over time (10–17). However, the risk factors and prognostic indicators of baseline variables for progression across non-IPF ILD subsets were not well established.

Therefore, in this single-center, retrospective study, we enrolled patients with non-IPF ILDs and assessed their clinical characteristics at baseline and outcomes over 1 year. We aimed to identify the features and potential risk factors associated with PPF in this population. Data on demographics, patient-reported outcomes, serial pulmonary function tests (PFTs), high-resolution computed tomography (HRCT) scores, and serum biomarkers were collected, and a multivariable logistic regression analysis was used to develop a prognostic nomogram model for predicting the likelihood of PPF in ILD patients.

## Methods

### Study design and populations

This was a retrospective study that aimed to identify non-IPF ILD patients who were at risk of developing a progressive fibrosing phenotype by searching electronic medical records.

Patients were treated at the China-Japan Friendship Hospital from January 2015 to December 2022. We included patients with a multidisciplinary diagnosis (pulmonologists, radiologists, and pathologists) of one of the following ILDs: connective tissue disease–associated interstitial lung disease (CTD-ILD), non-IPF idiopathic interstitial pneumonia (IIP), hypersensitivity pneumonitis (HP), sarcoidosis, and other ILDs. The assessment included clinical manifestation, specific history evaluation, smoking status, PFT changes, serological test results, HRCT, and lung biopsy, if needed. Patients with pulmonary embolism and decompensated heart failure were excluded (7–9). The Committee on Human Research at China-Japan Friendship Hospital approved the study design (2022-KY-166-1).

### Data collection

#### Demographics

Clinical predictors including age, sex, smoking status; symptom-based: modified Medical Research Council dyspnea score (mMRC); comorbidities: hypertension, diabetes, chronic obstructive pulmonary disease (COPD), combined pneumonia, and gastroesophageal reflux; and treatment history at baseline were documented.

#### Laboratory results

Biomarker predictors including the level of white blood cell (WBC), lymphocyte (LYM), lactic dehydrogenase (LDH), carbohydrate antigen 153 (CA153), carbohydrate antigen 125 (CA125), and carcinoembryonic antigen (CEA) at baseline were documented.

#### Pulmonary function tests

Pulmonary function testing (PFT) was performed in standard spirometry according to the American Thoracic Society (ATS)/European Respiratory Society (ERS) recommendations (3). Values were expressed as a percentage of predicted values. Forced vital capacity (FVC% pred), diffusion capacity for carbon monoxide (DLco% pred), and total lung capacity (TLC% pred) at baseline were recorded.

### Assessment of HRCT and calculation of HRCT score

HRCT data acquired at baseline were obtained at the end of inspiration and in the supine position using a variety of CT machines. The presence, extent, and distribution of CT findings were independently assessed by two experienced radiologists (Zhang and Han) and classified into grades 1 to 6 according to the classification of Ichikado et al. (18, 19): (I) Normal attenuation (spared area); (II) ground-glass attenuation (GGA) without traction bronchodilation or bronchiolectasis (TBE); (III) consolidation without TBE; (IV) GGA with TBE; (V) consolidation with TBE; and (VI) honeycombing. Of note, since the reticulations always overlapped with honeycombing, in this case, when the reticulations were presented, we attributed them to the honeycombing type.

Then, observers assessed the extent of all abnormalities to determine the percentage of lung parenchyma occupied by the disease. The lungs were divided into six regions (upper, middle, and lower on each side), and each zone was assessed separately. Scoring was based on the percentage of lung parenchyma showing evidence of abnormality and was estimated to be the nearest 10% of parenchymal involvement. The mean of the six lung regions found on each HRCT was the total percentage of lung involvement, calculated using the following formula to give a total HRCT score (20):

Overall HRCT score (%) = mean Normal attenuation score ^*^ 1+ mean GGA without TBE score ^*^ 2+ mean consolidation without TBE score ^*^ 3+ mean GGA with TBE score ^*^ 4+ mean consolidation with TBE score ^*^ 5+ mean honeycombing score ^*^ 6.

### PPF assessment

The primary outcome was ILD patients developing a progressive fibrosing phenotype, which met at least two of the following three criteria within the past year (4–6):

(I) Worsening of respiratory symptoms.
(II) Physiological evidence of disease progression (any of the following):

a. Absolute decrease in FVC (%) >5% predicted within 1 year of follow-up.
b. Absolute decrease in DLco (%) >10% predicted within 1 year of follow-up.

(III) Radiological evidence of disease progression (one or more of the following):

a. Increase in the extent or severity of TBE.
b. A new GGA with TBE.
c. A new fine reticulation.
d. Increased extent or increased coarseness of reticular abnormality.
e. New or increased honeycombing.
f. Increased lobar volume loss.

### Statistical analysis

Demographic and clinical characteristics between PPF patients and non-PPF patients were compared. Normally distributed data for continuous variables were expressed as mean ± standard deviation (SD), and non-normally distributed data were expressed as median (range). Categorical variables were expressed as percentages. Continuous variables were compared between groups using Student's *t*-test or the Mann-Whitney *U*-test, and categorical data were tested using the χ^2^ test. Some continuous predictors were divided into clinically meaningful categories, and univariate and multivariate logistic regression analyses were performed to identify the independent risk factors for ILD patients developing progressive phenotypes. Variables with a univariate relationship (*P* < 0.05) with PPF were entered into a multivariate logistic regression model, and a nomogram was plotted based on the results of multivariate analyses (*P* < 0.05) to construct predictive models. Calibration curves were depicted using the Kaplan–Meier method to evaluate the agreement between the nomogram prediction and actual observations, while the consistency of the model was determined using the Hosmer–Lemeshow test, with a *P* > 0.05 considered a good model fit. The obtained nomogram was compared with each single factor based on the area under the curve (AUC) using receiver operating characteristic curves (ROC). To assess the clinical utility of the predictive nomogram, a decision curve analysis (DCA) was performed by quantifying the net benefits of PPF at different threshold probabilities. *P* < 0.05 was statistically significant. R software (version 3.6.1) and customized code were used for the analysis.

## Results

**Figure 1:**
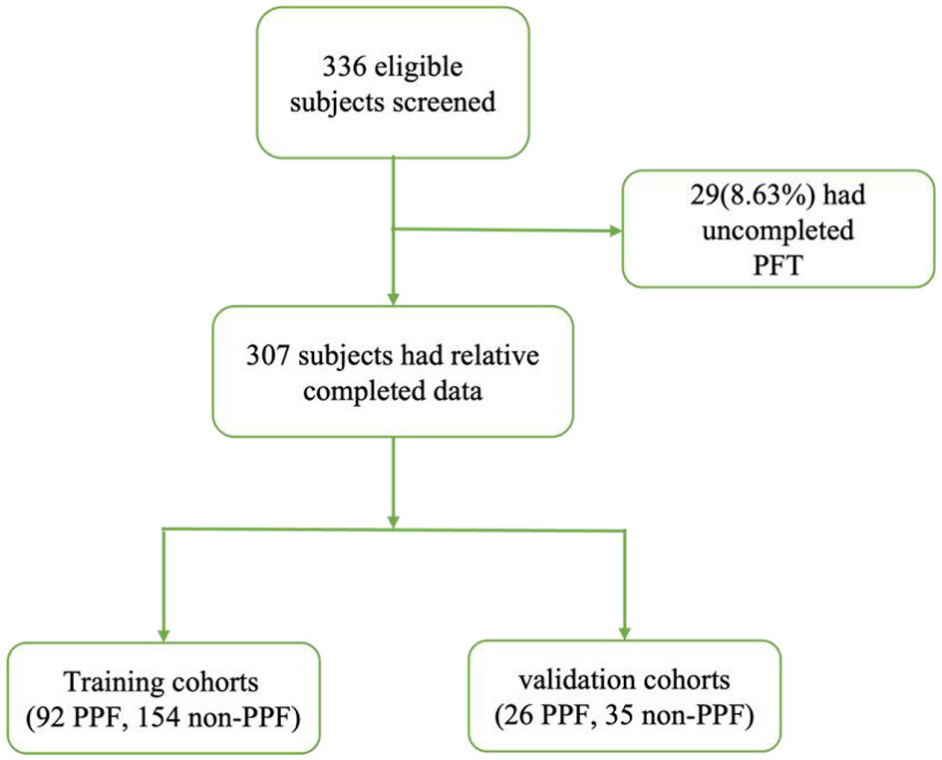
Reporting of observational studies in the epidemiology diagram.

We identified 336 patients from January 2015 to December 2022 (Figure 1). We excluded 29 patients who had missing data for at least one variable related to PFT. The final cohort consisted of 307 patients.

### Demographic features

Finally, 118/189 non-IPF ILD patients with or without progressive phenotypes were included in the final analysis. The demographic and clinical characteristics of all eligible patients are shown in Table 1. The mean age at inclusion was 62.26 years (SD, 22; *n* = 307), and 49.84% of patients were male. In total, 37.13% of patients had a history of smoking. Several patients had concurrent cardiorespiratory conditions, including chronic obstructive pulmonary disease (COPD) (34.85%) and combined pneumonia (24.76%). Other medical conditions occurring in patients included hypertension (34.85%), gastroesophageal reflux disease (46.91%), and diabetes (18.57%).

**Table 1:** Overall baseline demographic and clinical characteristics of patients with ILD.

**Variables**		**Cohort (*n* = 307)**	**PPF (*n* = 118)**	**non-PPF (*n* = 189)**	***P-*value**
**Demographics**					
Age, *n* (%)	< 60 years	103 (33.55%)	35 (29.66%)	78 (41.27%)	0.122
	≥60 years, ≤ 65 years	44 (14.33%)	22 (18.64%)	24 (12.7%)	
	>65 years	148 (48.21%)	61 (51.69%)	87 (73.73%)	
Male, *n* (%)		153 (49.84%)	62 (52.54%)	91 (77.12%)	0.483
Smoking status, *n* (%)	ex/current	114 (37.13%)	56 (47.46%)	58 (30.69%)	0.004^*^
	non	193 (62.87%)	62 (52.54%)	131 (69.31%)	
**Symptom-based**					
mMRC, mean (SD)		1.33 ± 1.13	2.31 ± 1.02	0.71 ± 0.66	< 0.01^*^
**Comorbidities**					
Hypertension, *n* (%)		107 (34.85%)	39 (33.05%)	68 (35.98%)	0.624
Diabetes, *n* (%)		57 (18.57%)	29 (24.58%)	28 (14.81%)	0.293
COPD, *n* (%)		28 (9.12%)	19 (16.1%)	9 (4.76%)	0.004^*^
Combined pneumonia, *n* (%)		76 (24.76%)	47 (39.83%)	29 (15.34%)	< 0.01^*^
Gastroesophageal reflux, *n* (%)		144 (46.91%)	64 (54.24%)	80 (42.33%)	< 0.01^*^
**Pulmonary function tests**					
FVC, % predicted, mean (SD)		76.71 ± 19.97	71.78 ± 19.84	79.79 ± 19.48	< 0.01^*^
DLco, % predicted, mean (SD)		70.9 ± 27.06	47.18 ± 18.85	85.7 ± 19.93	< 0.01^*^
TLC, % predicted, mean (SD)		68.59 ± 18.42	67.05 ± 14.68	69.55 ± 20.39	0.21
Index of oxygen, mean (SD)		377.59 ± 70.45	351.82 ± 76.22	393.67 ± 61.54	< 0.01^*^
**Radiography**					
HRCT score, mean (SD)		5.34 ± 7.22	10.18 ± 9.13	2.32 ± 3.02	< 0.01^*^
**Biomarker**					
WBC, *n* (%)	< 4 × 10^9^/L	23 (7.49%)	3 (2.54%)	20 (10.58%)	< 0.01^*^
	≥4 × 10^9^/L, ≤ 10 × 10^9^/L	249 (81.12%)	95 (80.5%)	154 (81.48%)	
	>10 × 10^9^/L	35 (11.4%)	20 (16.95%)	15 (7.94%)	
LYM, *n* (%)	< 0.4/L	6 (1.95%)	4 (3.39%)	2 (1.06%)	0.297
	≥0.4/L, ≤ 2/L	199 (64.82%)	78 (66.1%)	121 (64.02%)	
	>2/L	102 (33.22%)	36 (30.51%)	66 (34.92%)	
LDH, *n* (%)	< 100 U/L	0 (0%)	0 (0%)	0 (0%)	< 0.01^*^
	≥100 U/L, ≤ 300 U/L	272 (88.6%)	96 (81.36%)	176 (93.12%)	
	>300 U/L	35 (11.4%)	22 (18.64%)	13 (6.88%)	
CA153, *n* (%)	< 25 U/ml	177 (57.65%)	49 (41.53%)	128 (67.73%)	< 0.01^*^
	≥25 U/ml	130 (42.35%)	69 (57.98%)	61 (32.28%)	
CA125, *n* (%)	< 35 kU/L	211 (68.73%)	61 (51.69%)	150 (79.37%)	< 0.01^*^
	≥35 kU/L	96 (31.27%)	57 (49.15%)	39 (20.63%)	
CEA, *n* (%)	< 5 μg/L	219 (71.34%)	69 (57.98%)	150 (79.37%)	< 0.01^*^
	≥5 μg/L	88 (28.66%)	49 (41.53%)	39 (20.63%)	
**Initial treatment**					
Acetylcysteine, *n* (%)		74 (24.1%)	24 (20.33%)	50(26.46%)	0.27
Glucocorticoid, *n* (%)		140 (45.6%)	61 (51.69%)	79 (41.8%)	0.1
Nidanib, *n* (%)		8 (2.61%)	4 (3.39%)	4 (2.12%)	0.49
Pirfenidone, *n* (%)		21 (6.84%)	9 (7.63%)	12 (6.35%)	0.65
Cyclophosphamide, *n* (%)		24 (7.82%)	14 (11.86%)	10 (5.29%)	0.05
Cyclosporin, *n* (%)		3 (0.98%)	1 (0.85%)	2 (1.06%)	1
Mycophenolate, *n* (%)		10 (3.26%)	5 (4.24%)	5 (2.65%)	0.52

^*^Statistically significant subgroup effect sizes, ascertained as P_subgroup_ (χ^2^ test) < 0.05.

PPF, progressive pulmonary fibrosis; mMRC, modified Medical Research Council dyspnea score; COPD, chronic obstructive pulmonary disease; HRCT, high-resolution computed tomography; DLco% pred, diffusing capacity of carbon monoxide; FVC% pred, forced vital capacity; TLC% pred, total lung capacity; WBC, white blood cell; LYM, lymphocyte; LDH, lactic dehydrogenase; CA153, carbohydrate antigen 153; CA125, carbohydrate antigen 125; CEA, carcinoembryonic antigen. The pulmonary function data were expressed in liters and as a percentage of predicted (% pred).

There was no significant difference in drug use observed in the two cohorts. Almost all patients received at least one treatment. The most commonly used immunosuppressant therapy was glucocorticoid (45.6%), acetylcysteine (24.1%), cyclophosphamide (7.82%), mycophenolate mofetil (3.26%), and cyclosporin (0.98%). Few patients adopted antifibrotic therapy such as nidanib (2.6%) or pirfenidone (6.84%).

### Pulmonary function test and radiology

Patients had moderate lung function impairment at the time of enrollment in the whole cohort, with the mean DL_CO_% pred at baseline was 70.9%, FVC% pred was 76.71%, and TLC% pred was 68.59%. Significantly higher levels of pulmonary function tests excepted TLC% pred were observed in non-progressive patients (FVC% pred: 79.79% vs. 71.78%, *P* < 0.01; DLco% pred: 85.7% vs. 47.18%, *P* < 0.01; TLC% pred: 69.55% vs. 67.05%, *P*=0.21).

**Figure 2:**
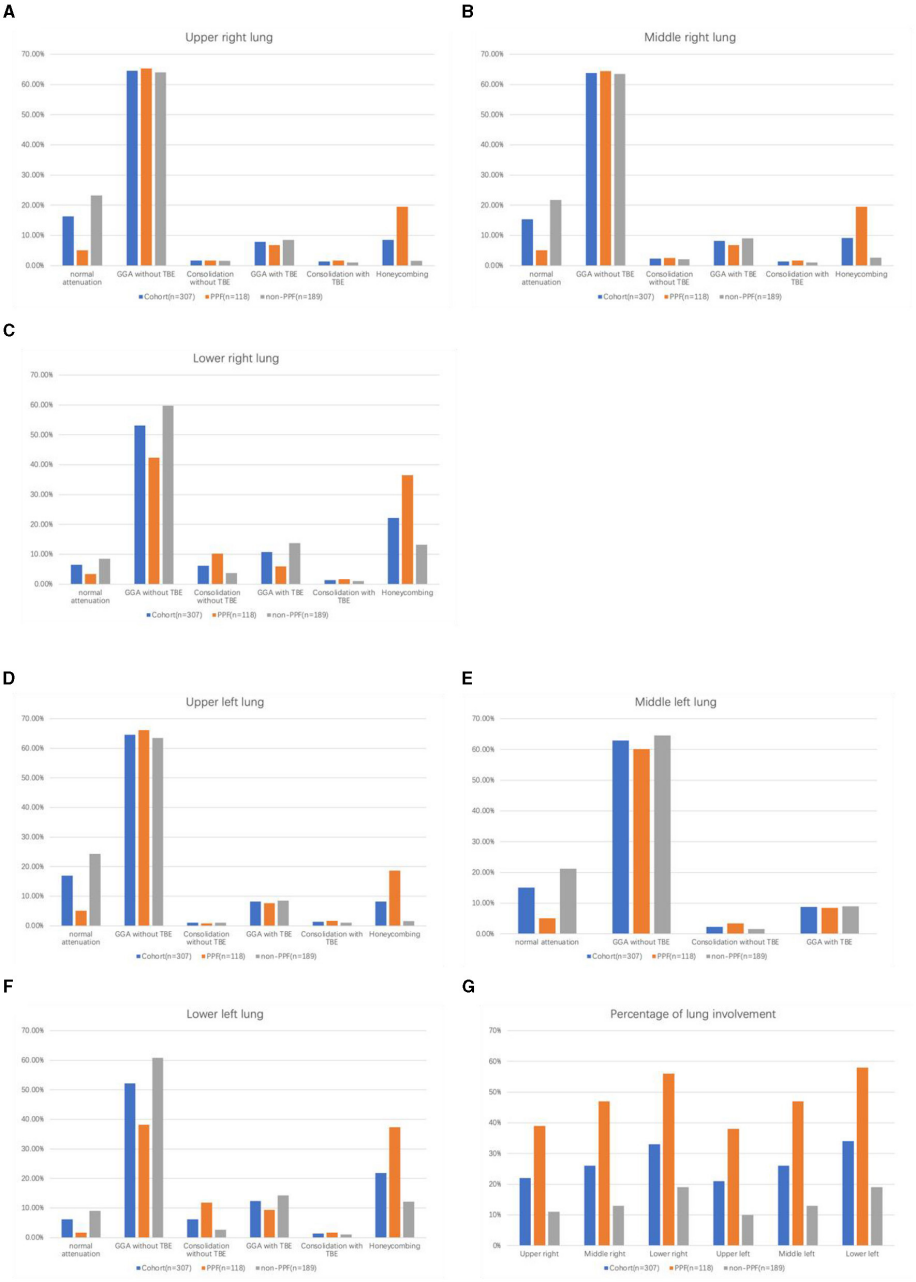
Histogram of the percentage of each abnormality on HRCT in six regions among PPF/non-PPF: **(A)** upper right lung, **(B)** middle right lung, **(C)** lower right lung, **(D)** upper left lung, **(E)** middle left lung, **(F)** lower left lung, and the total percentage of lung parenchyma occupied by the disease in six regions among PPF/non-PPF **(G)**.

**Figure 3:**
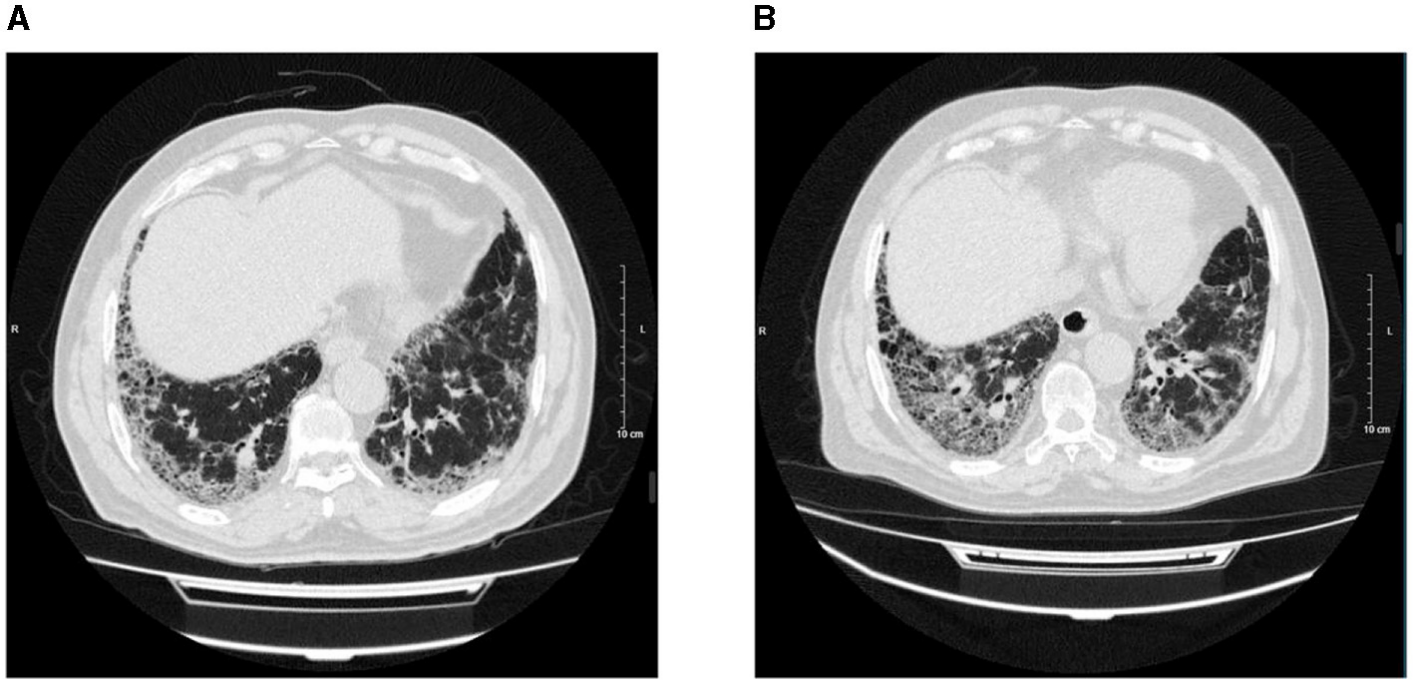
Axial HRCT images of a 75-year-old male, on 29 October 2021 **(A)** and 4 October 2022 **(B)** showed peripheral and basilar predominant progressive pulmonary fibrosis with the increased extent of reticulation, traction bronchiectasis, and honeycombing.

The range of the overall HRCT score was 5.34 (SD, 7.22). There was a statistically significant difference in the HRCT scores between PPF (mean, 10.18 [SD, 9.13]) and non-PPF (mean, 2.32 [SD, 3.02]). Honeycombing in the six zones of the lung was more frequent in PPF (*P* < 0.01), especially in the lower zones of the lung. GGA without TBE was the most prominent HRCT pattern in the two cohorts, having a higher incidence for the non-progressive group in the lower zones of both the left and right lung, with statistics of 38.14% vs. 60.85%, *P* < 0.01 and 42.37% vs. 59.79%, *P* < 0.01. No significant differences in consolidation with or without TBE or GGA with TBE were observed between the two cohorts. PPF patients showed a higher percentage of lung involvement in six zones of the lung (48% vs. 14%, *P* < 0.01). Furthermore, compared with the upper and middle zones, the lower zones observed more fibrosis in the two groups (*P* < 0.01). Details of the evaluation of HRCT are elucidated in Figure 2. Additionally, we provided axial HRCT images of a 75-year-old male who experienced PPF (Figure 3).

### Non-IPF ILD subtypes

The final cohort had 307 patients with ILD other than IPF, including 120 (39.09%) CTD-ILD, 115 (37.46%) non-IPF IIP, 35 (11.4%) HP, 6 (1.95%) Sarcoidosis, and 31(28.97%) other ILD. Except for unclassifiable-ILD (10.17% vs. 24.34%, *P* < 0.01), no significant difference in each subtype of ILD was observed during the two cohorts (Table 2). Furthermore, compared with CTD-ILD, we found a higher level of baseline PFT in non-IPF IIP (FVC% pred: 72.37% vs. 78.88%; TLC%: 66.49% vs. 70.8%; DLco% pred: 68.96% vs. 73.74%).

**Table 2:** Clinical ILD diagnoses documented on the case report form.

	**Cohort (*n* = 307)**	**PPF (*n* = 118)**	**non-PPF (*n* = 189)**	***P-*value**
CTD-ILD, *n* (%)	120 (39.09%)	52 (44.07%)	68 (35.98%)	0.19
ASS-ILD, *n* (%)	33 (10.75%)	13 (11.02%)	20 (10.58%)	1
RA-ILD, *n* (%)	28 (9.12%)	15 (12.71%)	13 (6.87%)	0.1
SS-ILD, *n* (%)	26 (8.47%)	6 (5.08%)	20 (10.58%)	0.14
IIM-ILD, *n* (%)	11 (3.58%)	7 (5.93%)	4 (2.12%)	0.11
ANCA-ILD, *n* (%)	9 (2.93%)	4 (3.39%)	5 (2.65%)	0.74
MCTD-ILD, *n* (%)	8 (2.61%)	3 (2.54%)	5 (2.65%)	1
MPA-ILD, *n* (%)	3 (0.98%)	2 (1.69%)	1 (0.53%)	0.56
AOSD-ILD, *n* (%)	1 (0.33%)	1 (0.85%)	0 (0%)	0.38
AS-ILD, *n* (%)	1 (0.33%)	1 (0.85%)	0 (0%)	0.38
Non-IPF IIP, *n* (%)	115 (37.46%)	34 (28.81%)	81 (42.86%)	0.02^*^
Unclassifiable ILD, *n* (%)	58 (18.89%)	12 (10.17%)	46 (24.34%)	< 0.01^*^
iNSIP, *n* (%)	57 (18.25%)	22 (18.64%)	35 (18.52%)	1
HP, *n* (%)	35 (11.4%)	14 (11.86%)	21 (11.11%)	0.86
Sarcoidosis, *n* (%)	6 (1.95%)	2 (1.69%)	4 (2.12%)	1
Other ILD	31 (28.97%)	16 (13.56%)	15 (7.94%)	0.12
IPAF, *n* (%)	15 (4.89%)	10 (8.47%)	5 (2.65%)	0.05
Exposure-related ILD, *n* (%)	6 (1.95%)	1 (0.85%)	5 (2.65%)	0.41
PAP, *n* (%)	5 (1.63%)	2 (1.69%)	3 (1.59%)	1
Medication-induced, *n* (%)	3 (0.98%)	1 (0.85%)	2 (1.06%)	1
After chemotherapy, *n* (%)	1 (0.33%)	1 (0.85%)	0 (0%)	0.38
FPF, *n* (%)	1 (0.33%)	1 (0.85%)	0 (0%)	0.38

^*^Statistically significant subgroup effect sizes are ascertained as P_subgroup_ (χ^2^ test) < 0.05.

ILD, interstitial lung disease; CTD-ILD, connective tissue disease–associated interstitial lung disease; ASS, antisynthetase syndrome; RA-ILD, rheumatoid arthritis-ILD; SS-ILD, systemic sclerosis-ILD; IIM-ILD, idiopathic inflammatory myopathy-ILD; ANCA-ILD, anti-neutrophil cytoplasm antibodies-ILD; MCTD-ILD, mixed connective tissue disease-associated ILD; MPA-ILD, microscopic polyangiitis-ILD; ASOD-ILD, adult-onset still disease-ILD; AS-ILD, ankylosing spondylitis-ILD; HP, hypersensitivity pneumonitis; iNSIP, idiopathic non-specific interstitial pneumonia; COP, cryptogenic organizing pneumonia; IPAF, interstitial pneumonia with autoimmune features; PAP, pulmonary alveolar proteinosis; DIP, desquamative interstitial pneumonitis; FPF, familial pulmonary fibrosis.

### Risk factors for ILD progression and a predictive model

The results of univariate and multivariate logistic regression analyses of PPF are presented in Table 3. Before performing the multivariate logistic regression, a diagnostic test for multicollinearity was conducted by the variance inflation factor (VIF), which showed no multicollinearity (VIF < 10). Finally, the results from multivariate logistic regression analysis showed that combined pneumonia (OR=4.57, 95% CI, 2.54–18.43, *P* < 0.01), low baseline DLco% pred (OR = 0.91, 95% CI, 0.89–0.93, *P* < 0.01), high mMRC marks (OR = 6.34, 95% CI, 4.23–10.14, *P* < 0.01), and high HRCT score (OR = 1.38, 95% CI, 1.25–1.54, *P* < 0.01) were independent risk factors associated with PPF.

**Table 3:** Univariate and multivariate analysis of predictors of PPF.

	**Univariable analysis**		**Multivariable analysis**	
	**OR (95% CI)**	***P*** **-value**	**OR (95% CI)**	***P-*** **value**
Smokers, *n* (%)	2.03 (1.19, 3.49)	< 0.01		
COPD, *n* (%)	3.45 (1.36, 9.52)	0.01		
Combined pneumonia, *n* (%)	4.66 (2.54, 8.78)	< 0.01	4.57 (1.24, 18.43)	0.02
mMRC, mean ± SD	6.34 (4.23, 10.14)	< 0.01	4.9 (2.8, 9.5)	< 0.01
FVC% pred, mean ± SD	0.98 (0.97, 1.00)	< 0.01		
DLco% pred, mean ± SD	0.91 (0.89, 0.93)	< 0.01	0.92 (8.93, 0.95)	< 0.01
Index of oxygenation, mean ± SD	0.99 (0.99, 0.99)	< 0.01		
WBC, *n* (%)	2.61 (1.39, 5.19)	< 0.01		
LDH, *n* (%)	4.42 (1.8, 11.93)	< 0.01		
CA153, *n* (%)	2.62 (1.55, 4.51)	< 0.01		
CA125, *n* (%)	3.96 (2.25, 7.07)	< 0.01		
CEA, *n* (%)	2.33 (1.31, 4.17)	< 0.01		
HRCT, mean ± SD	1.38 (1.25, 1.54)	< 0.01	1.22 (1.07, 1.42)	< 0.01

OR, odds risk; 95% CI, 95% confidence interval; SD, standard deviation; PPF, progressive pulmonary fibrosis; COPD, chronic obstructive pulmonary disease; mMRC, modified Medical Research Council dyspnea score; FVC% pred, forced vital capacity; DLco% pred, diffusing capacity of carbon monoxide; WBC, white blood cell; LDH, lactic dehydrogenase; CA153, carbohydrate antigen 153; CA125, carbohydrate antigen 125; CEA, carcinoembryonic antigen; HRCT, high-resolution computed tomography.

### Performance of the nomogram

**Figure 4:**
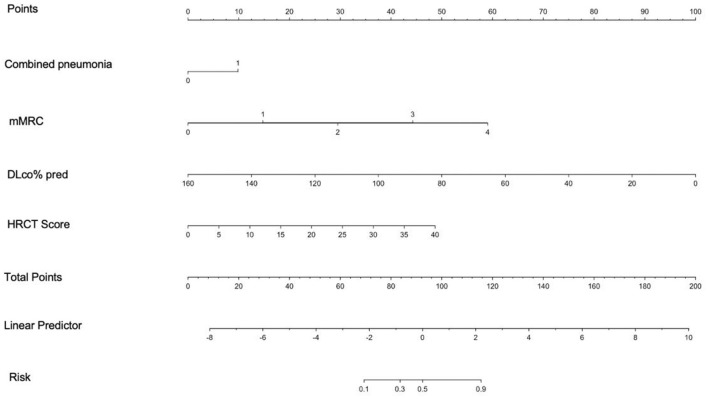
Nomogram derived from multivariable analysis for predicting progressive fibrosis. The points from each of the four components of the nomogram: Combined pneumonia (0 = 0 point, 1 = 9.3 points), mMRC (0 = 0 point, 1 = 12.45 points, 2 = 24.9 points, 3 = 37.35 points, 4 = 49.8 points), DLco% pred (points = 100-0.625* DLco% pred), and HRCT score (points = 1.02* HRCT score), are the predicted progressive fibrosis obtained from each scale by referring to the corresponding value.

A nomogram was constructed from the results of the multivariable analysis to determine the total score and probability of PPF in a patient from the training cohort (*n* = 246) and internally validated in 61 patients (Figure 4).

**Figure 5:**
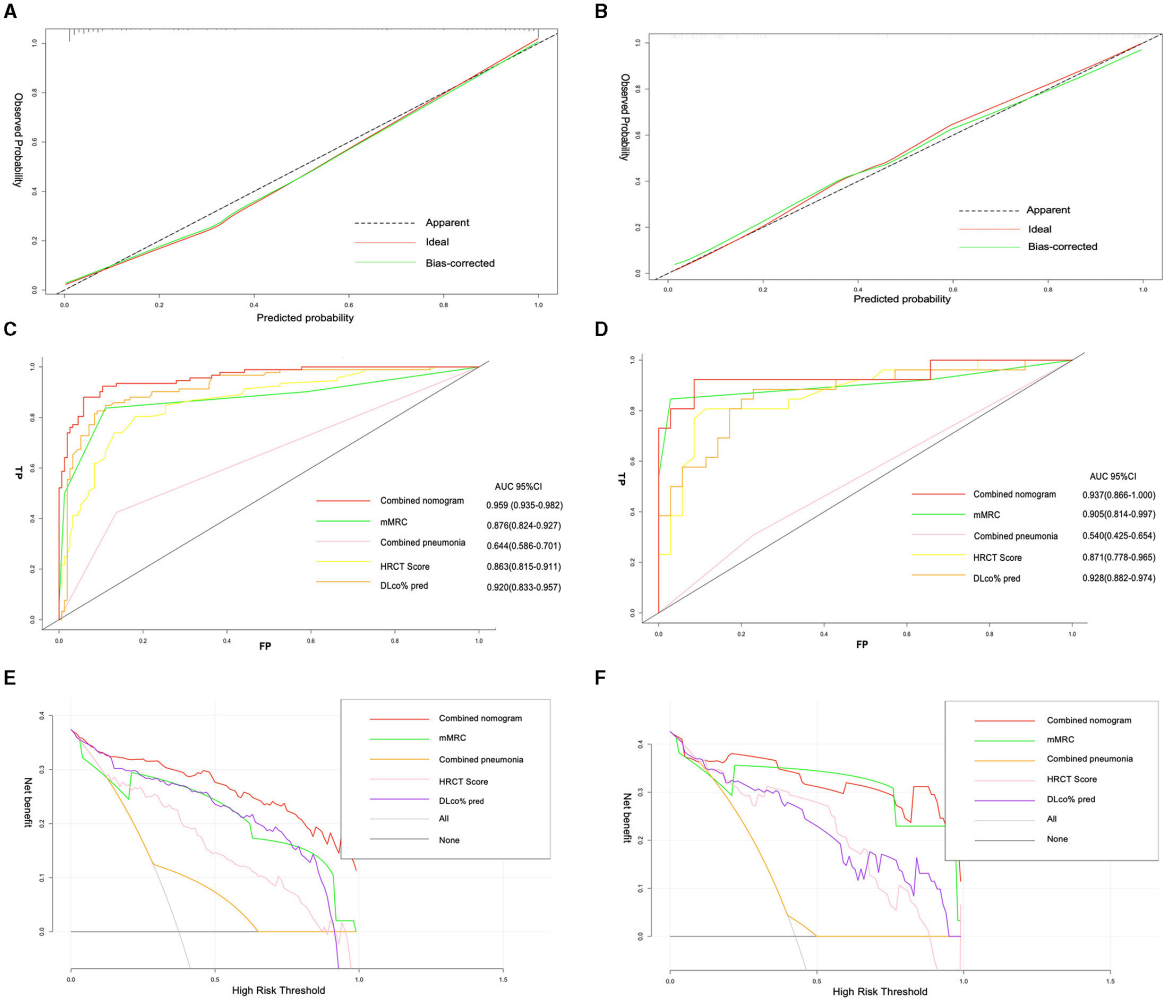
Calibration plots of nomogram showing predicted progressive fibrosis against actual progressive pulmonary fibrosis in the training set **(A)**, and validation set **(B)**. The AUC of the combined model, mMRC, combined pneumonia, HRCT score, and DLco% pred in the training set **(C)**, and validation set **(D)**; decision curve analysis for the combined model, mMRC, combined pneumonia, HRCT score, and DLco% pred in the training set **(E)**, and validation set **(F)**.

The calibration plots of the training and validation groups showed high accuracy and agreement between the predicted nomogram and real observations. The Hosmer–Lemeshow test confirmed the good fit of the nomogram (*P* = 0.86). The ROC curve was used to compare the nomogram model with each single risk factor from the multivariate logistic regression. The AUC of the nomogram model was 0.96 (95% CI, 0.94–0.98), and the AUCs of the single risk factors were 0.88 (95% CI, 0.82–0.93) for mMRC, 0.64 (95% CI, 0.59–0.7) for combined pneumonia, 0.86 (95% CI, 0.82–0.91) for HRCT score, and 0.92 (95% CI, 0.83–0.96) for DLco% pred. The DCA curve showed that the combined model had more clinical benefit and performance than each single factor in both training and validation sets (Figure 5).

### Validation in different ILD subtypes

Calibration plots showed that the nomogram that predicted the fibrotic progression in CTD-ILD was closer to the actual rates than non-IPF IIP. The AUCs of the combined model were 0.96 (95% CI, 0.92–0.99) in the CTD-ILD cohort and 0.94 (95% CI, 0.87–1) in the non-IPF IIP cohort, which was significantly higher than the AUC obtained for each variable in the model. The decision curve showed that the threshold probability of a patient or doctor using a combined model to predict progressive fibrosis in ILD would be more beneficial than each signal factor in two main cohorts (Supplementary Figure 1).

## Discussion

The concept of PPF arose from the observation that a substantial proportion of patients with non-IPF ILDs developed a progressive fibrosis phenotype similar to IPF, with a rapid decline in lung function and early mortality (4, 21). In this study, we developed and validated a nomogram model for predicting the risk of PPF in non-IPF ILD patients using a retrospective study. We found that combined pneumonia, low baseline DLco% pred, high mMRC marks, and a high HRCT score were significant predictors of PPF, and this model showed good performance in predicting the incidence of PPF, which enabled physicians to identify patients whose disease was likely to progress using baseline information.

Previous studies have proposed various predictors of survival in IPF or progression in specific types of ILDs based on changes in variables over time (10–13). However, the risk factors and prognostic indicators of baseline variables for progression across non-IPF ILD subsets were not well established. Following the example of Ley et al. (22), who constructed a multidimensional GAP (gender [G], age [A], and two lung physiology variables [P] [FVC% and DLco%]) index and staging system using variables that were commonly measured in clinical practice to predict mortality in IPF, we also developed and validated a model that used physiologic, radiologic, and symptomatic variables to identify patients with non-IPF ILDs who will develop a progressive fibrotic pattern. We finally derived a nomogram based on combined pneumonia, baseline DLco% pred, mMRC scores, and HRCT scores to predict ILD progression. Our study also showed that these predictors had different impacts on PPF, with DLco% pred, mMRC, and HRCT scores having more weight than combined pneumonia.

First, we found that smoking status was associated with a higher incidence of progression, as non-smokers had a lower odds ratio than ex/current smokers (OR=2.03, 95% CI, 1.19–3.49). This may be related to the smoking-induced changes in cellular function that contributed to the pathogenesis of IPF (11, 23). Although previous research suggested that reflux/dysphagia symptoms resulting from esophageal motility dysfunction and chronic microaspiration were strong predictors of FVC% pred decline over time (24) and could cause persistent alveolar epithelial damage and accelerate pulmonary fibrosis, we did not find a multivariate link between gastroesophageal reflux and PPF in this research. Moreover, no significant differences were observed in baseline age, sex status, hypertension, diabetes, TLC% pred, the level of LYM, or initial treatment between PPF and non-PPF patients.

Second, this study documented a strong association between combined pneumonia and PPF (OR = 4.57, 95% CI, 1.24–18.43, *P* = 0.02), which could be explained by the increased infiltration of pro-inflammatory and pro-fibrotic cells that induced the production of pro-fibrotic cytokines and progressive remodeling of the fibrotic tissue (25), indicating that the early stages of fibrotic disease may be characterized by complex inflammatory events involving both the innate and adaptive immune systems.

Third, our data corroborated recent studies indicating that the decline of the HRCT score should serve as the linchpin of PPF criteria (OR = 1.22, 95% CI, 1.07–1.42), as it could assess and quantify the range of parenchymal abnormalities, including ground-glass opacities, consolidation, and honeycombing (26). These features added prognostic information to the histopathological diagnosis, as shown in previous studies of IPF (27), RA-ILD (28), SSc-ILD (29), chronic HP (30), pulmonary sarcoidosis (31), and unclassifiable ILD (32). Furthermore, the HRCT score used in this study had been previously documented to be an independent prognostic factor in patients with acute respiratory distress syndrome (ARDS) secondary to pneumonia, as well as AE-IPF and acute interstitial pneumonia by Ichikado et al. (18, 19). In addition to our findings, their findings also supported the hypothesis that this HRCT scoring system was useful in determining the prognosis of patients with acute and progressive fibroproliferative lung disease. Of note, we observed the base of the lower lungs was the most involved lung field in non-IPF ILD, and GGA without TBE was frequently found there. Moreover, Lee et al. (33) proposed the hypothesis, elucidating that specific radiological features such as honeycombing and traction bronchiectasis were associated with a worse prognosis (33), we confirmed part of it, as honeycombing was the most prominent HRCT pattern in PPF patients in our study, while the distribution of traction bronchiectasis was similar in both groups. Other CT distributions did not show a significant correlation with the patient outcome.

Next, our data suggested that a low baseline of DLco% pred should be the strongest and most consistent predictor of PPF incidence in this non-IPF ILD cohort, which was in line with previous studies (34, 35). However, we acknowledged that isolated low DLco% could sometimes reflect a worsening of pulmonary vascular disease rather than a progression of ILD (36). Therefore, PPF should be considered when patients have a low DLco% with concurrent FVC decline (26). A lower baseline FVC% was also an established predictor of mortality in patients with progressive fibrosing ILDs, as evidenced by numerous studies spanning IPF (10, 11), RA-ILD (12), SSc-ILD (29), and chronic HP (13). In this study, we also demonstrated that PPF patients had significantly lower mean baseline FVC% pred than stable ILD patients (71.78% vs. 79.79%, *P* < 0.01). However, we did not observe a statistical difference between the two groups in TLC% pred.

The mMRC, a validated symptom questionnaire for various lung diseases, was used in this study to assess quality of life, disease severity, and prognosis. According to our results, the mMRC score was one of the strongest predictors of PPF. We did not use it as an independent feature, as it could introduce ascertainment bias without objective evidence of lung function decline or fibrosis progression on HRCT.

Finally, the mechanisms and differences of PPF in different ILD subtypes were poorly understood. Previous studies indicated that patients in each ILD subgroup had similar clinical phenotypes of reduced lung function, worsening symptoms, impaired quality of life, and increased mortality (37–42). Pugashetti et al. (43) confirmed a >10% decline in relative FVC and strongly predicted decreased survival in non-IPF ILD patients across different cohorts. In our study, CTD-ILD (39.09%) and non-IPF IIP (37.46%) were the most common patterns in our ILD cohort. Except for unclassifiable ILDs, there was no significant difference between the PPF and non-PPF groups. The AUC of the combined nomogram was 0.96 (95% CI, 0.92–0.99) in the CTD-ILD cohort and 0.94 (95% CI, 0.87–1) in the non-IPF IIP cohort, showing favorable accuracy and efficacy in the distinct ILD subsets. Though the sample size was insufficient to detect the efficacy of nomograms in different disease subgroups, our results supported the hypothesis that PPF may result from a common mechanism of fibrosis in various ILDs, regardless of the initial cause or association. Furthermore, our data also agreed with recent studies that suggested phenotypic variability in ILD subtypes even with PPF criteria. Without homogenization of the PPF phenotype, calibration plots showed that CTD-ILD progression rates were closer to the observed rates, while PPF in non-IPF IIP was underestimated, and a higher level of baseline PFT was observed in non-IPF IIP than CTD-ILD. These results may contribute to the hypothesis that fibrosing ILDs with a progressive phenotype had some similarities but differences also existed, and this situation may also be due to the overrepresentation of one ILD subtype.

## Limitations

Our study has some limitations that should be acknowledged. First, this was a single-centered, retrospective study, which was suboptimal compared to prospective trials and might have biased patient selection. Next, due to the retrospective nature of this study, the duration of follow-up varied by patient and cohort, precluding the assessment of each PPF feature over a standardized time frame. Furthermore, this study involved a small number of patients, and the number of each subtype of patients with ILD enrolled was relatively small, which meant it was not possible to draw definite conclusions regarding enrichment in certain subgroups. Prospective studies with larger samples were needed to further validate our findings. Second, we relied on the mMRC questionnaire for symptomatic worsening, which may have introduced some degree of subjective bias but was similar to the methodology used in a recent PPF clinical trial. Third, our predictive model was not validated externally by more multicenter studies with enlarged patient cohorts, leading to a lack of generalizability of this model. Fourth, since we assessed HRCT according to Ichikado et al. (18, 19), which was conducted in 2002 and 2006. Due to the increasing depth of modern studies on the image of PF-ILD, the reference value of older research methods was limited. Moreover, although reticulations were rarely observed in the cohorts enrolled in this study, when reticulations were present, we attributed them to the honeycombing type, neglecting that reticulation may be present without honeycombing, which may introduce some selection bias. Moreover, although baseline pulmonary function tests were useful for predicting prognosis, changes over time may improve predictive power, but we did not document the changes in PFT. Finally, clinical and laboratory data from recent studies have reported that serum levels of KL-6 are elevated in a variety of ILDs, including IPF and collagen vascular disease-associated interstitial pneumonia (14–17). While most patients included in this research had absent data associated with KL-6, large prospective research with comprehensive indicators should be done to further verify and optimize the predictive model.

## Conclusion

We developed and validated a prognostic nomogram model for predicting the risk of PPF in ILD patients based on four clinical predictors: baseline DLco% pred, complicated pneumonia, mMRC scores, and HRCT scores. The nomogram showed good discrimination and calibration in both the training and validation cohorts, and it outperformed every single predictor in terms of accuracy and efficiency. We believe the nomogram could help clinicians stratify ILD patients into different risk groups and tailor their management accordingly. Our study also characterized some features of ILD patients who may develop a progressive fibrosis phenotype, such as CTD-ILD and non-IPF IIP. However, our study had some limitations, such as the retrospective design, the single-center setting, and the potential selection bias. Furthermore, longitudinal studies are needed to confirm and optimize the predictive model in different populations and settings. The nomogram is a useful tool for risk assessment and decision-making, but it is not a substitute for clinical judgment or individualized care.

## Data availability statement

The original contributions presented in the study are included in the article/Supplementary material, further inquiries can be directed to the corresponding author.

## Ethics statement

The studies involving humans were approved by the Committee on Human Research at China-Japan Friendship Hospital approved the study design (2022-KY-166-1). The studies were conducted in accordance with the local legislation and institutional requirements. The participants provided their written informed consent to participate in this study.

## Author contributions

J-MG: Writing – original draft, Writing – review & editing. J-JF: Writing – review & editing. S-YX: Data curation, Methodology, Writing – review & editing. M-YJ: Methodology, Supervision, Writing – review & editing. G-LH: Visualization, Writing – review & editing.
