# Supplementary material for: Risk factors for progression of pulmonary fibrosis: a single-centered, retrospective study
Source: Front Med (Lausanne). 2024 Feb 7;11:1335758. doi: 10.3389/fmed.2024.1335758 (PMC10879408; doi:10.3389/fmed.2024.1335758)
Supplement: Supplementary file 1 [file Data_Sheet_1.docx]

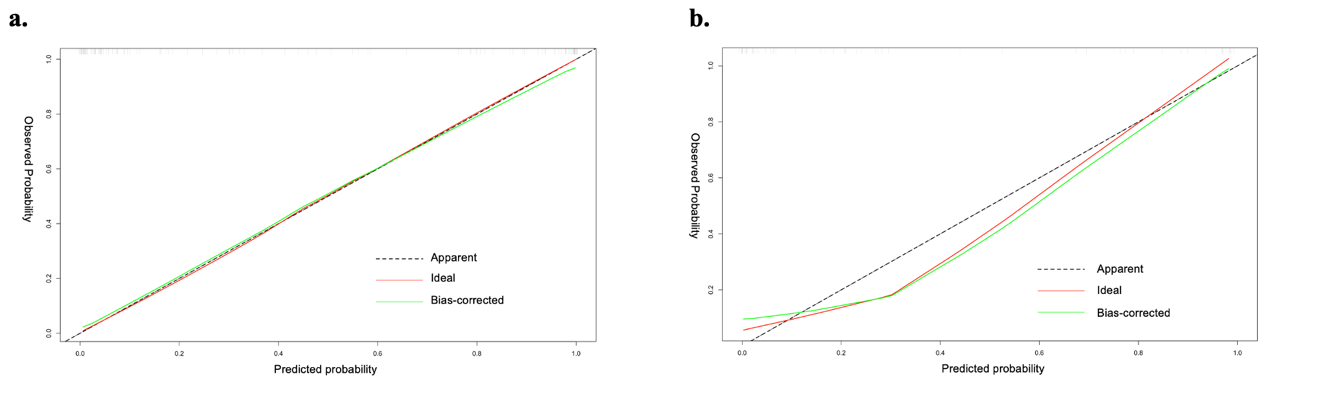


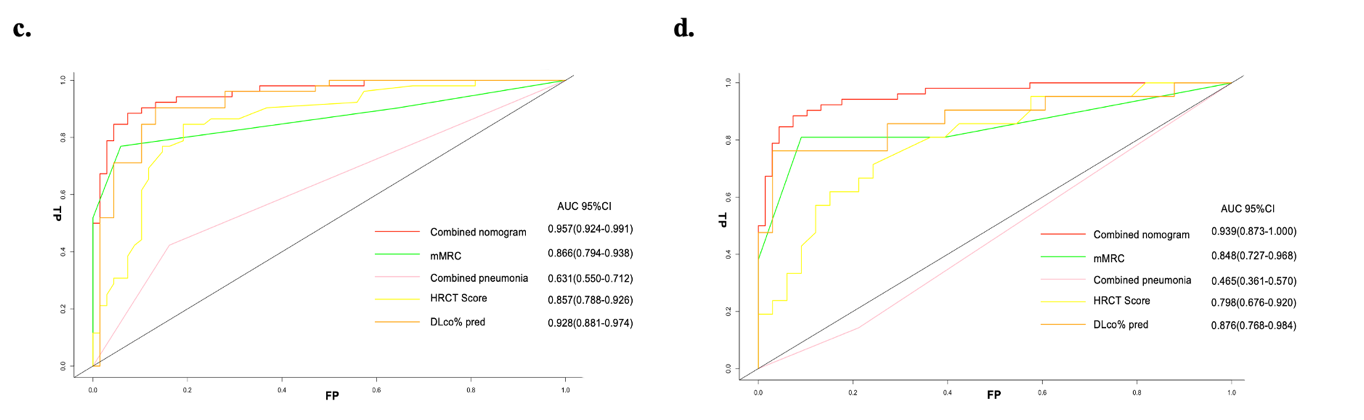


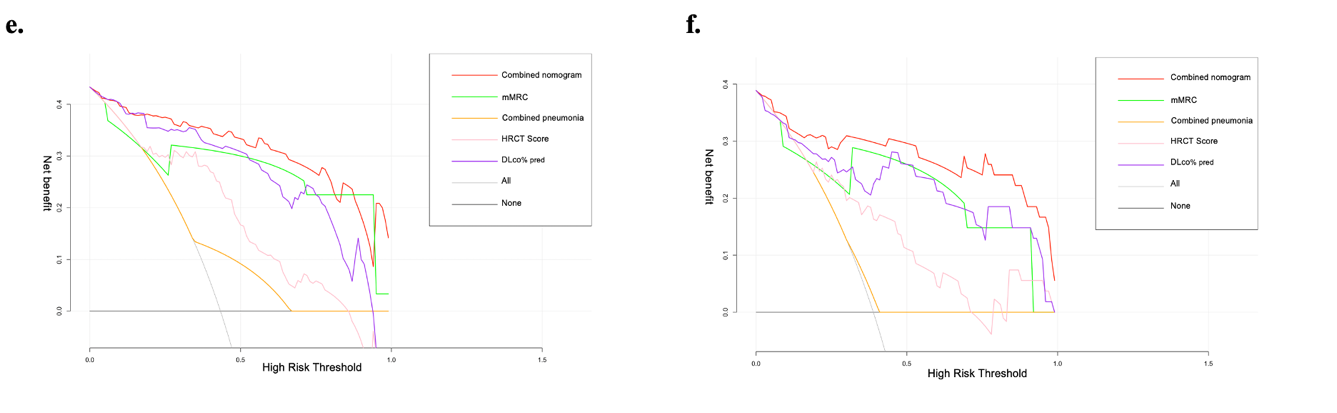


S Fig 6. Calibration plots of nomogram showing predicted progressive fibrosis by against actual progressive pulmonary fibrosis in the CTD-ILD cohort (a), and non-IPF IIP cohort (b). The AUC of combined model, mMRC, complicated pneumonia, HRCT score, and DLco% pred in the CTD-ILD cohort (d), and non-IPF IIP cohort; Decision curve analysis for the combined model, mMRC, complicated pneumonia, HRCT score, and DLco% pred in the CTD-ILD cohort (g), non-IPF IIP cohort.
